# Beating the Odds: Is Mental Health at Stake for High-Achieving Children in Poverty in the ABCD Study?

**DOI:** 10.1192/j.eurpsy.2023.1162

**Published:** 2023-07-19

**Authors:** S. Pacheco, M. E. Ellwood-Lowe, S. A. Bunge

**Affiliations:** Psychology, University of California, Berkeley, Berkeley, United States

## Abstract

**Introduction:**

Childhood family income is a powerful predictor of academic achievement and mental health. Here, we ask whether children living in poverty–those whose family incomes are not sufficient to meet their material needs–who beat the odds by succeeding academically are subsequently either protected from, or more at risk for, internalizing disorders. Prior research indicates that children in poverty with better academic performance and more depressive symptomatology tend to have higher temporal coupling between lateral frontoparietal network (LFPN; supports executive functions) and Default Mode Network (DMN; supports internally-directed thought) than lower-performing children in poverty, in direct contrast to the pattern observed for children above poverty. Thus, an open question is whether this pattern of connectivity adaptive for children in poverty has maladaptive long-term consequences, particularly for mental health.

**Objectives:**

In this pre-registered study, we analyzed concurrent data from 8,091 children (1,307 in poverty) in the ABCD study at baseline (ages 9-10y). We performed linear mixed effects models to investigate whether both higher LFPN-DMN connectivity and grades are linked to more internalizing symptoms concurrently, and whether this differs for children above and below poverty.

**Methods:**

We performed linear mixed effects models to investigate whether both higher LFPN-DMN connectivity and grades are linked to more internalizing symptoms concurrently, and whether this differs for children above and below poverty.

**Results:**

We found that higher grades were associated with fewer internalizing symptoms for both children above and below poverty; this association was stronger for children below poverty. In addition, LFPN-DMN connectivity showed a significant negative correlation with internalizing symptoms at this age. However, when looking at internalizing symptoms separately - that is, anxiety/depression, withdrawal/depression, and somatic symptoms - we found that higher LFPN-DMN connectivity for children below poverty was associated with higher withdrawal/depression symptoms, but fewer somatic symptoms, pointing to a dissociation in what pattern of brain connectivity is most adaptive for the development of internalizing symptoms vs. physical health. These somatic symptoms highlight potential maladaptive consequences of resilience for children growing up in unequal structural conditions.

**Conclusions:**

This research has important implications for supporting children in poverty by illuminating mechanisms for, and potential maladaptive consequences of, their resilience in academic contexts.

**Disclosure of Interest:**

None Declared

